# New Insights in Paediatric Dermatopathology

**DOI:** 10.3390/dermatopathology8040056

**Published:** 2021-12-07

**Authors:** Sylvie Fraitag

**Affiliations:** Paediatric Dermatopathology Unit, Department of Pathology, Hôpital Necker-Enfants Malades, APHP, 75015 Paris, France; sylvie.fraitag@aphp.fr

**Keywords:** paediatric dermatopathology, childhood melanoma, auto-inflammatory disorders, nodules, neonates, infants, bullous eruption in infancy, panniculitis, congenital cystic masses of the neck, congenital cysts, pseudo-malignancy, subcutaneous spindle cell neoplasms, ichthyoses, venous malformations, tuberous sclerosis complex

Paediatric dermatology is an expanding subspeciality. This is well illustrated by the growing number of books and articles published on this subject in recent years [1,2,3].

Paediatric dermatopathology is also a relatively recent and less explored niche. There are many skin conditions common in both adults and children. However, some skin disorders are mostly observed in childhood, and some are restricted to paediatric age. Paediatric dermatopathology covers a wide range of disorders including genodermatoses, cutaneous and sub-cutaneous tumours, and inflammatory disorders.

Over the last decade, I have witnessed the expansion of paediatric dermatology alongside paediatric dermatopathology. Currently, paediatricians and paediatric dermatologists are no longer reluctant to routinely perform a cutaneous biopsy in a young child, even for a vascular lesion. As a result, dermatopathologists are more and more called upon to deal with skin biopsies in neonates, infants, and children. This was certainly not the case when I started my practice in paediatric dermatopathology.

This Special Issue covers a subset of paediatric dermatological conditions in which a great deal of knowledge has been achieved in recent times, thanks to the development of new immunomarkers, the application of molecular biology, and the introduction of new classification system(s). This Special Issue is addressed to dermatologists and pathologists interested in paediatric dermatopathology.

The difficult and complex subject of ichthyoses is brilliantly covered by Metze, D. and Traupe, H. [4]. An accurate and rapid diagnosis of a hereditary keratinization disorder is important not only for the identification of associated diseases of internal organs in syndromic forms, but it can be instrumental for genetic counselling and therapy [5,6]. It is demonstrated well in this article that these cornification disorders have different histological patterns that can allow their better recognition before genetic testing.

The onset of blisters in infancy is always a source of great concern for both parents and physicians. These blistering rashes can be caused by a wide range of benign or severe life-threatening disorders of different aetiologies, including infections, genetics, or autoimmune conditions. The underlying cause has to be determined urgently and a skin biopsy is often required and effective in achieving a rapid and precise diagnosis, and to guide the appropriate management of the baby. In this article, Leclerc-Mercier, S. illustrates in detail the different causes of blistering rashes in the neonatal period [7]. In her review, the author also provides practical instructions to the readers on how to deal with skin biopsy(s) in blistering disorders, e.g., choice of the lesion and site of the biopsy, choice of fixators, when to freeze the sample, and/or perform electron microscopy. This article is very well written and is a practical guide for dermatologists as well as pathologists from a very experienced physician in blistering disorders.

Autoinflammatory diseases are a group of emerging and growing entities defined by aberrant, antigen-independent activation of the innate immune signalling pathways [8]. The dermatopathologist’s role is crucial as autoinflammatory disorders can be suspected upon cutaneous histopathological examination. Kolivras, A. et al. provide a very comprehensive overview of these complex disorders and offer practical clues that can help the clinicians in the differential diagnosis [9]. The authors describe three major histopathological patterns seen in autoinflammatory skin diseases, i.e., the “neutrophilic” pattern, the “vasculitic” pattern, and the “granulomatous” pattern. The recognition of these different patterns can facilitate the diagnosis of monogenic forms versus complex multigenic diseases.

Very little has been published on the histological aspect of skin lesions in Tuberous Sclerosis Complex (TSC). The gap has been filled by the excellent article published by Cascarino, M. and Leclerc-Mercier, S. [10]. The authors provide the reader with a comprehensive clinico-pathological overview of the different skin lesions observed in TSC, focusing on hypomelanotic lesions and cutaneous hamartomas. Helpful clues are given.

The diagnosis of cutaneous and subcutaneous spindle cell neoplasms in children is always challenging because of the potential therapeutic and prognostic implications. In addition to the well-known dermatofibrosarcoma protuberans and infantile fibrosarcoma, new entities have been described in the last decade, often with the help of cytogenetics. This comprehensive article from Drabent, P. focuses on these new entities, and their similarities and differences, such as lipofibromatosis and lipofibromatosis-like neural tumour, or plexiform myofibroblastoma and plexiform fibrohistiocytic tumour. In addition, the authors attempt to unify and simplify some confusing and close entities such as fibroblastic connective tissue nevus, medallion-like dermal dendrocyte hamartoma, or plaque-like CD34-positive dermal fibroma [11].

Cutaneous melanomas are very rare in children and their diagnosis is always challenging. The knowledge of the different types of melanomas in children has greatly improved over the last few years, mainly thanks to cytogenetics. This excellent review from de La Fouchardière, A. et al. illustrates with clarity the complexity of paediatric melanocytic malignant lesions and the need for an accurate histological classification to ensure the best clinical management, being the prognosis linked to the melanoma subtype [12].

The diagnostic of panniculitis is challenging at any age, especially in children. This subject is particularly difficult as panniculitis includes a wide range of diseases, with some entities being undoubtedly rare and difficult to recognize. Adults and children share some entities. However, several forms of panniculitis are mostly observed in childhood, or are even restricted to the paediatric age group and particularly the neonatal period. New entities have been recognized recently, mostly linked to auto-inflammation or auto-immunity [13], whereas others, obscure entities, such as Rothmann–Makai syndrome or Weber–Christian disease, are no longer considered to be specific entities. There are very few up-to-date reviews summarizing the different causes of panniculitis in children and detailing their clinical and pathological particularities. The article from Moulonguet, I. [14] is the most recent and comprehensive review on this difficult subject.

The term “pseudomalignancy” covers a large, heterogeneous group of diseases characterized by a benign cellular proliferation, hyperplasia, or infiltrate that resembles a true malignancy, either clinically or histologically. Several inflammatory skin diseases or benign proliferations in children can mimic malignant neoplasms. In this very comprehensive article, Menzinger, S. et al. [15] review the different entities observed in children, which are all traps into which the pathologist can fall. To my knowledge, there is no comparable review in the literature; this is why it seems important to me to have this article at hand when dealing with paediatric cutaneous biopsies.

Congenital cystic masses of the neck are very frequently encountered in paediatrics and their accurate classification may be challenging. Furthermore, this subject is very little and poorly described in dermatopathology books. The aim of these very experienced authors [16] is to describe clinical, radiological, and pathological findings of all the different entities, in order to provide the clinician and the pathologist with the relevant characteristics required for proper management.

Neonatal conditions with multiple cutaneous nodules have a poor prognosis and thus require a prompt diagnosis. A skin biopsy should always be part of the initial examination because the lesion’s histology will guide the recommendations for further investigations and the patient’s clinical management. This review summarizes the clinical and pathological features of the various disorders that may manifest at birth as multiple skin lesions, and provides the reader with numerous clinical and histological photos [17].

Our knowledge in vascular anomalies has greatly increased in recent years, thanks to the development of cytogenetics with the identification of signalling pathways and genetic mutations responsible for the development of vascular tumours/malformations [18,19]. Venous malformations (VM) represent a heterogeneous group of lesions presenting in the skin, soft tissue, and sometimes, the viscera. The recognition of relevant histopathological features, along with strict clinicopathological correlation, is essential to properly classify these lesions. This very comprehensive article, written by the best specialists in vascular anomalies [20], provides an overview of the clinicopathological features and molecular alterations of venous malformations (VM) in childhood. This review covers common VMs such as blue rubber bleb nevus syndrome, glomuvenous malformation, cerebral malformation, familial intraosseous vascular malformation, and verrucous venous malformation.
